# Pemphigus foliaceus transforming to pemphigus vulgaris: a case report

**DOI:** 10.3389/fmed.2025.1707841

**Published:** 2026-01-02

**Authors:** Mandlin Abdulaziz Almousa, Navya Kuchibhotla, Lama Alabdulaaly, Marcus Couey, Vikki Noonan, Herve Sroussi

**Affiliations:** 1 Division of Oral Medicine and Dentistry, Brigham and Women's Hospital, Boston, MA, United States; 2 Department of Oral Medicine, Infection and Immunity, Harvard School of Dental Medicine, Boston, MA, United States; 3 Oral Medicine and Oral Oncology Service, Dana-Farber Cancer Institute, Boston, MA, United States; 4 Department of Basic Sciences, College of Dentistry, Princess Nourah Bint Abdulrahman University, Riyadh, Saudi Arabia; 5 Maxillofacial Surgery and Diagnostic Sciences Department, College of Dentistry, King Saud bin Abdulaziz University for Health Sciences, Riyadh, Saudi Arabia; 6 Dental Services Department, Ministry of the National Guard Health Affairs, Riyadh, Saudi Arabia; 7 King Abdullah International Medical Research Center, Riyadh, Saudi Arabia; 8 Department of Oral and Maxillofacial Surgery, Boston University Henry M. Goldman School of Dental Medicine, Boston, MA, United States; 9 Division of Oral Pathology, Boston University Henry M. Goldman School of Dental Medicine, Boston, MA, United States

**Keywords:** pemphigus vulgaris, pemphigus foliaceous, transformation, vesiculobullous, corticosteroids, rituximab

## Abstract

Pemphigus represents a family of acantholytic autoimmune vesiculobullous diseases. It is classified into multiple subtypes, the two most common of which are pemphigus vulgaris and pemphigus foliaceus. The transition between these two subtypes is uncommon. In this report, we discuss a unique case of biopsy-proven pemphigus foliaceus transitioning to pemphigus vulgaris.

## Introduction

The pemphigus groups of diseases are rare, chronic, autoimmune vesiculobullous conditions which may result in significant morbidity and mortality. Multiple subtypes of pemphigus diseases have been identified, however; pemphigus vulgaris (PV) and pemphigus foliaceous (PF) are the two main forms. PV is more common and accounts for 60%−90% of all pemphigus cases while PF accounts for 10%−30% (1, 2). PV is divided into two subtypes: mucosal-dominant PV and mucocutaneous PV. In mucosal-dominant PV, desmoglein 3 (Dsg3; abundantly present in mucosal tissues) is targeted by autoantibodies, causing extensive mucosal lesions. In mucocutaneous PV, autoantibodies are directed against both desmoglein 1 (Dsg1) and Dsg3, thus resulting in skin and mucosal lesions (1, 3, 4). In contrast, in PF, autoreactive B cells produce autoantibodies targeting Dsg1, which is predominantly expressed throughout the epidermis, with higher concentrations in the superficial layers. Hence, lesions in PF are typically confined to the skin. Transition between these two pemphigus subtypes has been rarely reported, with most documented cases involving a shift from PV to PF. In contrast, transformation from PF to PV is exceptionally uncommon (5, 6). We present a case of biopsy-proven PF transforming to PV, a rare phenomenon.

## Case presentation

**Figure 1:**
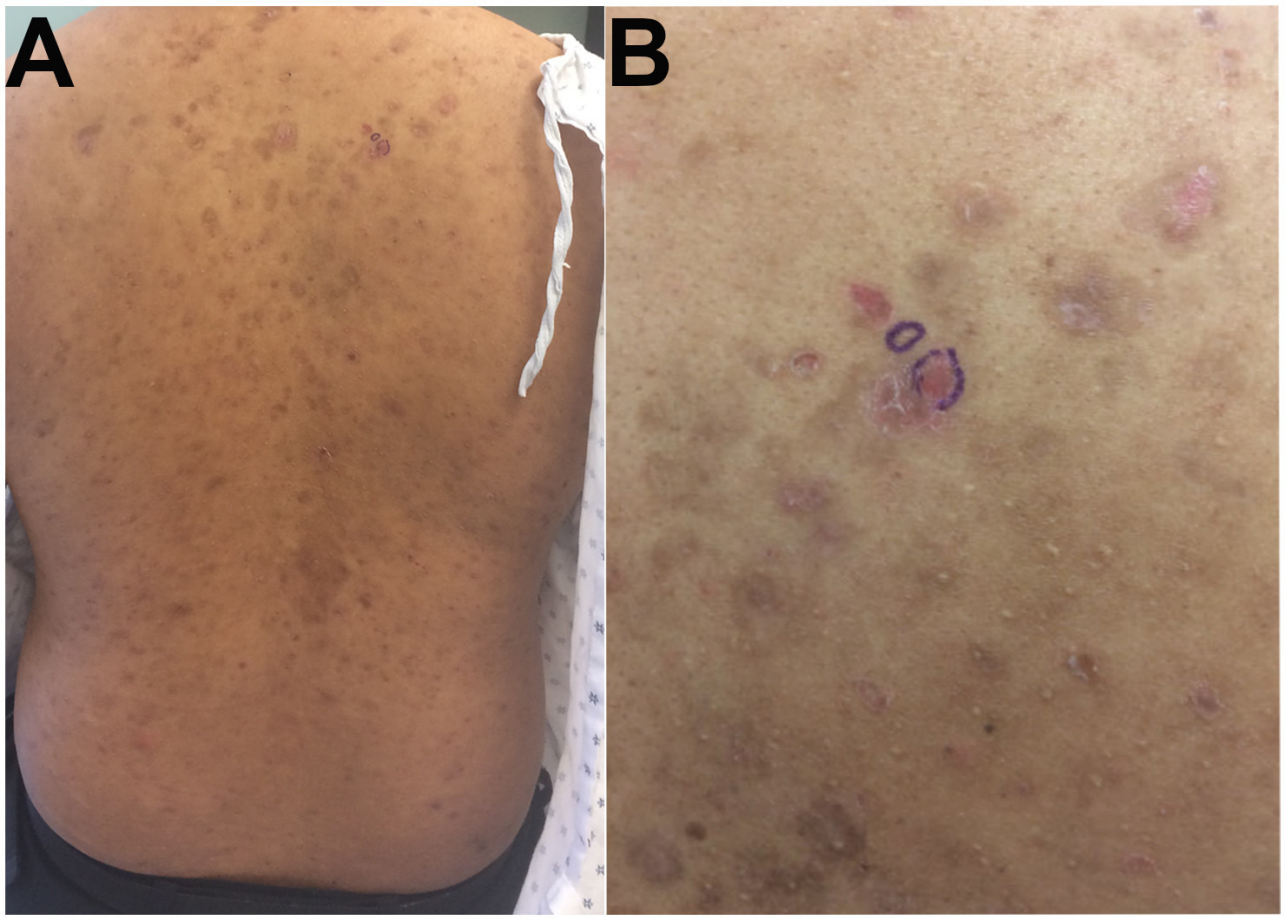
**(A)** Widespread scaly brown-purple macules scattered on the back, **(B)** biopsied area is circled in blue.

**Figure 2:**
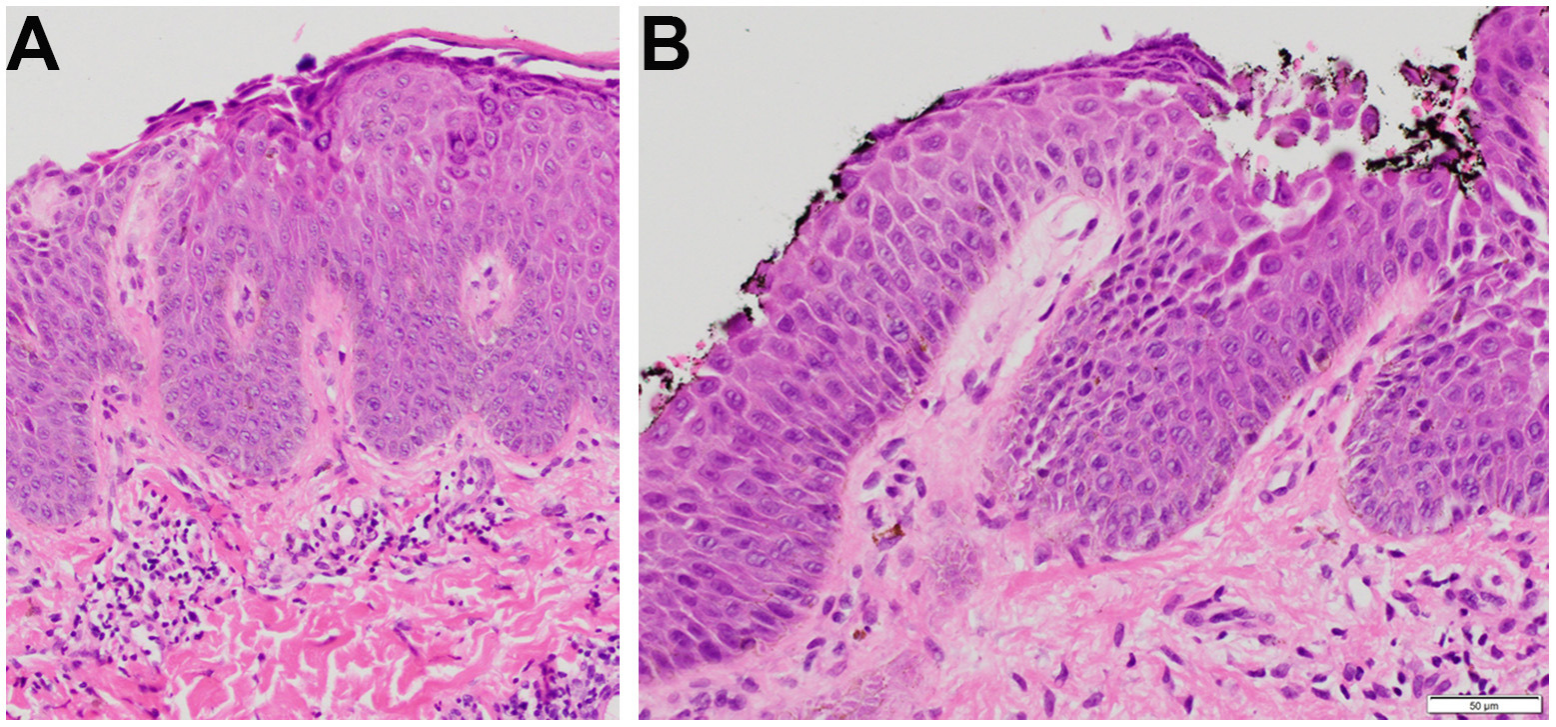
Histopathologic findings from the punch biopsy specimen obtained from the skin of the back. **(A)** Subcorneal clefting (100×), **(B)** Intraepidermal clefting and acantholysis (200×).

A 28-year-old male of mixed Haitian and Japanese descent, with no significant past medical history, presented to the dermatology clinic in October 2022 with pruritic, hyperpigmented, scaly macules on the back (Figure 1), chest, and scalp, without oral involvement. A punch biopsy showed acantholysis in the upper third of the epidermis on hematoxylin and eosin (H&E) staining (Figure 2) consisted with PF.

In December 2022, he sought a second opinion at another dermatology clinic, where biopsies again demonstrated intracorneal and intragranular acantholysis with superficial lymphocytic infiltration. Direct immunofluorescence (DIF) revealed intercellular epidermal deposition of IgG with weak C3 and no IgA or IgM deposits, confirming the diagnosis of PF.

Serologic testing in January 2023 revealed elevated anti-Dsg1 (148 U/mL; negative < 18, positive > 36) and anti-Dsg3 in the indeterminate range (23 U/mL; negative < 19, positive > 37). Testing for BP180 and BP230 antibodies was negative, helping to exclude bullous pemphigoid. Although low-level anti-Dsg3 reactivity was present, the overall clinical, histopathologic, and serologic findings were most consistent with PF at that stage.

The patient was started on mycophenolate mofetil 1 g twice daily (BID), doxycycline 100 mg BID, and topical corticosteroids for the skin and scalp, including clobetasol 0.05% shampoo, fluocinonide 0.01% oil, and triamcinolone 0.1% ointment. A single intravenous immunoglobulin (IVIg) infusion was administered but discontinued due to headaches.

By June 2023, repeat serology demonstrated persistently elevated anti-Dsg1 (131 U/mL) and a further increase in anti-Dsg3 (58 U/mL), raising concern for transition from PF to PV. Shortly after, the patient developed new-onset intermittent oral sloughing that progressively worsened, marking the first evidence of mucosal involvement. He was started on a dexamethasone 0.05% oral rinse, with no reported improvement despite consistent use.

**Figure 3:**
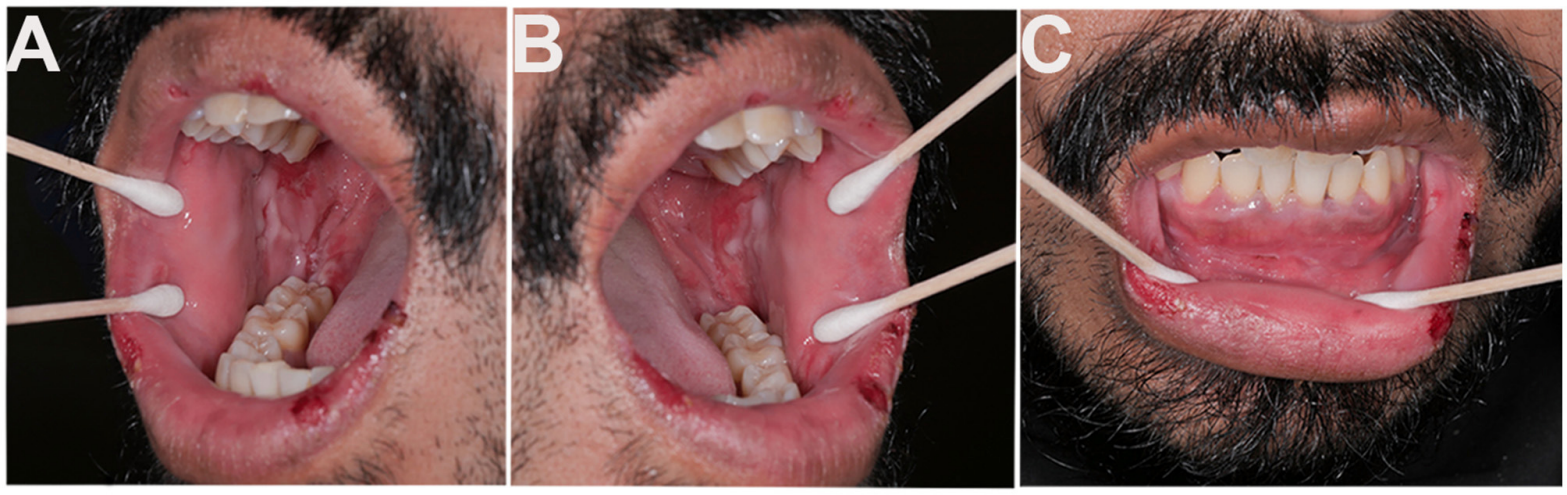
Scattered ulcerations with sloughing and marked erythema on the **(A)** right buccal mucosa and **(B)** left buccal mucosa. **(C)** Ulcerations and sloughing involving the lower lip mucosa and vestibule, with hemorrhagic crusting and ulcerations also affecting the lip vermilion.

In October 2023, he presented to the Oral Medicine Clinic with significant oral pain and reduced quality of life. Examination revealed ulcerations of the ventrolateral tongue, lips, and bilateral buccal mucosa with erythema, sloughing, and hemorrhagic crusting (Figure 3). The dexamethasone rinse was replaced with clobetasol 0.05% solution, and tacrolimus 0.1% ointment for the lips. Unfortunately, he continued to experience worsening symptoms, with persistent oral lesions and worsening skin disease characterized by blisters and erosions involving the lower extremities. He was started on a prednisone taper (initiated at 60 mg daily, reduced by 20 mg every 28 days) for extensive mucocutaneous involvement, resulting in partial improvement.

**Figure 4:**
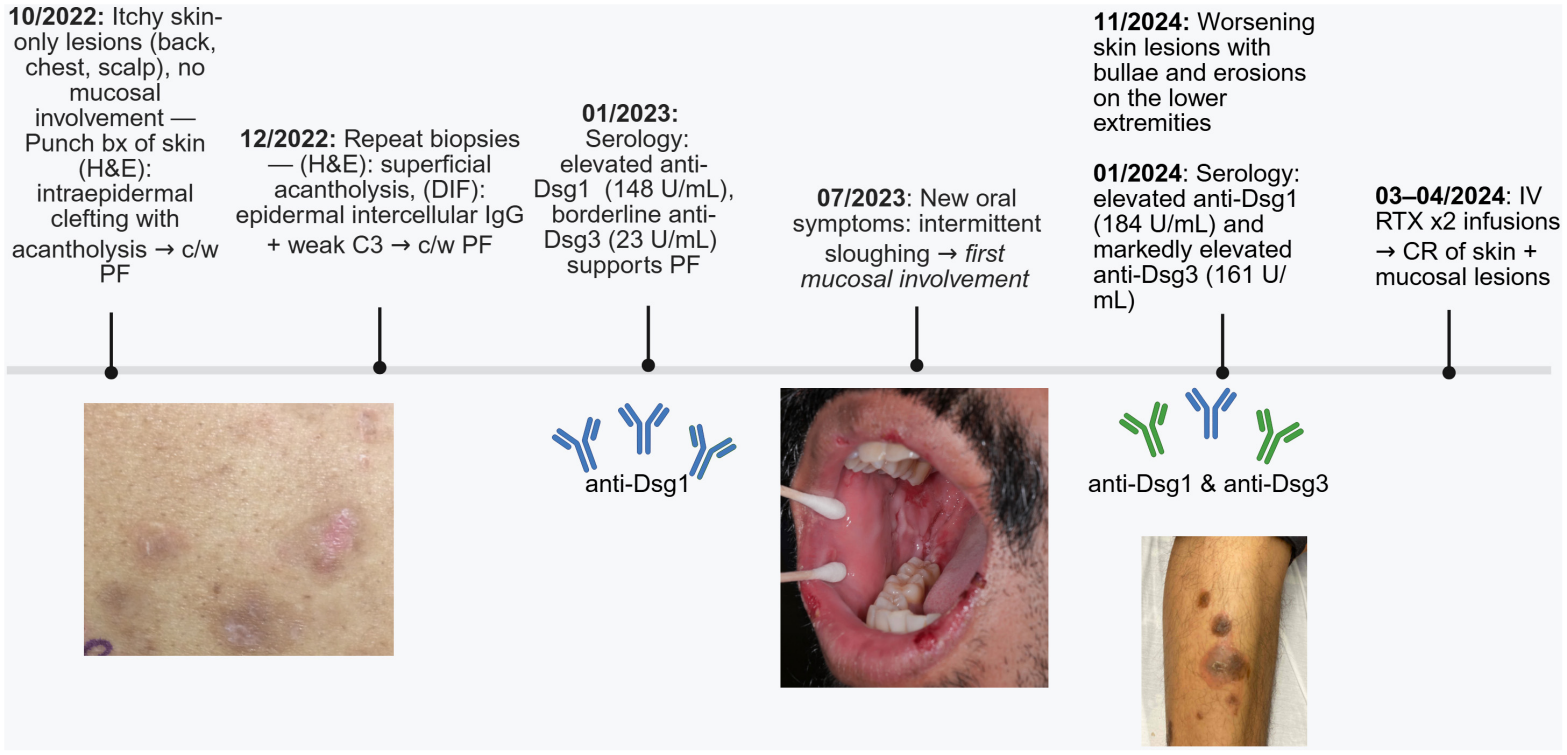
Timeline summarizing the patient's disease course. bx, biopsy; H&E, hematoxylin and eosin; DIF, direct immunofluorescence; IgG, immunoglobulin G; C3, complement component 3; PF, pemphigus foliaceus; anti-Dsg, anti-desmoglein; PV, pemphigus vulgaris; IV, intravenous; RTX, rituximab; CR, complete resolution; c/w, consistent with. Created in BioRender. Almousa, M. (2026) https://BioRender.com/b6xxbid.

Repeat serology in January 2024 revealed a further increase in anti-Dsg1 (184 U/mL) and a marked rise in anti-Dsg3 (161 U/mL). This phenotypic change, with progression from superficial skin-limited lesions to mucocutaneous blistering and erosive disease and accompanying serologic conversion from anti-Dsg1–dominant to anti-Dsg3 co-dominant antibodies, confirmed transition to mucocutaneous PV. The patient received two infusions of rituximab 1 g each in March and April 2024 while continuing prednisone 30 mg daily. Two weeks after the second infusion, he reported complete resolution of lesions. Mycophenolate and doxycycline were discontinued. In June 2024 during his dermatology follow-up, he remained symptom-free on 5 mg prednisone. A timeline of the case presentation can be found in Figure 4.

## Discussion

Although uncommon, cases of both PF to PV transformation and vice versa have been documented, with the former less frequently observed (5, 7–9). The progression from PF to PV may be explained by “epitope spreading,” wherein chronic inflammation and tissue damage expose previously hidden antigens like Dsg3, leading to the generation of new pathogenic autoantibodies (10, 11).

Treatment strategies for PV and PF aim to achieve remission and long-term maintenance (12) and therapy is guided by disease severity. For moderate to severe PV, recent clinical evidence shows that rituximab is effective across different dosing strategies: the standard 1 g dose, a lower 500 g dose, and even an ultra-low dose of 100 mg, all administered as 2 infusions, 2 weeks apart. These regimens led to high remission rates (92.3%−100%) with >99% B-cell depletion and steroid-sparing effects. Ultra-low dose RTX demonstrated the lowest cost and adverse effects profile, making it a viable first-line modality (13). When rituximab is contraindicated or unavailable, corticosteroids may be combined with steroid-sparing agents such as azathioprine or mycophenolate mofetil. In mild PV, corticosteroids (prednisone 0.5–1 mg/kg/day) with or without immunosuppressants are often effective, although rituximab may also be considered for early disease control and steroid-sparing benefits. For PF, mild cases are often treated with topical corticosteroids with or without oral dapsone (50–100 mg/day). Systemic corticosteroids are introduced if lesions are extensive. Rituximab is generally reserved for severe or refractory cases of PF (14).

## Conclusion

Transformation between the subtypes of pemphigus is rare, with reports of PF transforming into PV being particularly uncommon in the literature. This underscores the importance of closely monitoring patients with pemphigus and conducting necessary diagnostic tests to detect any potential progression or transformation of the disease over time to appropriately direct management, as necessary.

## Data Availability

The original contributions presented in the study are included in the article/supplementary material, further inquiries can be directed to the corresponding author.
